# Characterization of Annexin V Fusion with the Superfolder GFP in Liposomes Binding and Apoptosis Detection

**DOI:** 10.3389/fphys.2017.00317

**Published:** 2017-05-19

**Authors:** Abdul Qader Abbady, Aya Twair, Bouthaina Ali, Hossam Murad

**Affiliations:** ^1^Department of Molecular Biology and Biotechnology, Atomic Energy Commission of SyriaDamascus, Syria; ^2^Department of Animal Biology, Faculty of Sciences, Damascus UniversityDamascus, Syria

**Keywords:** apoptosis, annexin V, *sf*GFP, fusion proteins, liposomes, exosomes, phospholipids, SPR

## Abstract

Programed cell death is a critical and unavoidable part of life. One of the most widely used markers for dying cells, by apoptosis or pyroptosis, is the redistribution of phosphatidylserine (PS) from the inner to the outer plasma membrane leaflet. Annexin V protein is a sensitive and specific probe to mark this event because of its high affinity to the exposed PS. Beyond that, annexin V can bind to any PS-containing phospholipid bilayer of almost all tiny forms of membranous vesicles like blood platelets, exosomes, or even nanostructured liposomes. In this work, recombinant human annexin V was produced as a fusion with a highly fluorescent superfolder derivative of the green fluorescent protein (*sf*GFP) in *Escherichia coli*. The fusion protein(*sf*GFP-ANXV, 64 kDa), annexin V (ANXV, 40 kDa), and *sf*GFP (27 kDa) were separately produced after cloning their encoding genes in pRSET plasmid, and all proteins were expressed in a soluble form, then purified in high yields because of their *N*-terminal 6× His tag (~150 mg of pure protein per 1 L culture). Superiority of this fluorescent fusion protein over fluorescein-conjugated annexin V was demonstrated in binding to phospholipids (and their liposomes), prepared from natural sources (soya bean and egg yolk) that have different content of PS, by using different methods including ELISA, dot-blotting, surface plasmon resonance, and flow cytometry. We also applied fluorescent annexin V in the detection of apoptotic cells by flow cytometry and fluorescent microscopy. Interestingly, *sf*GFP-ANXV fusion was more sensitive to early apoptotic stressed HeLa cells than fluorescein-conjugated-ANXV. This highly expressed and functional *sf*GFP-ANXV fusion protein provides a promising ready-to-use molecular tool for quantifying liposomes (or similarly exosomes) and detecting apoptosis in cells.

## Introduction

From the quiet turnover of worn out cells and cellular components to the inflammatory death that warns of infection, cell death is involved in countless areas of scientific research. Programed cells death is a genetically directed process of cell self-destruction, which usually accompanied by different characteristic morphological and biochemical changes (Vermeulen et al., 2005). It ensures the proper development of multicellular organisms by maintaining tissue homeostasis and protects the organism through the elimination of unwanted (through apoptosis) or infected (through pyroptosis) cells (Fink and Cookson, 2005). During the early stage of apoptosis, cells lose their phospholipid membrane asymmetry and expose phosphatidylserine (PS) at the cell surface. Generally, annexins are a family of highly conserved proteins that specifically bind anionic phospholipids, including PS, in a calcium-dependent manner (Fatimathas and Moss, 2010; Lizarbe et al., 2013). One of the annexin family that has extracellular presence in addition to intracellular localization is annexin V (or A5) (Hmila et al., 2010). While all annexins bind to PS and calcium, they vary in their affinity for PS. In the presence of calcium ions, annexin V binds selectively to the exposed PS residues at the outer leaflet of the plasma membrane of apoptotic cells with nanomolar affinity (Kdt 0.5–7 nM), while it shows minimal binding to other negatively charged phospholipids like phosphatidylcholine and sphingomyeline (van Engeland et al., 1998; Vuchelen et al., 2009).

Annexin V, a 35 kDa protein, was first described by Reutelingsperger et al., as a vasculature-derived protein with great anticoagulant properties (Reutelingsperger et al., 1985). Ever since, annexin V became a universal marker of apoptosis and a versatile tool to inspect the changes at the level of cell membranes (Lizarbe et al., 2013; Wang et al., 2015). Because of its implication in Ca^2+^ signaling, expression of annexin V was found to be in strict correlations with several diseases, including cancer, autoimmune disorders, and diabetes (Fatimathas and Moss, 2010), and it may be considered as a specific marker for Alzheimer's disease (Sohma et al., 2013). Annexin V has been used in the detection of blood platelets microparticles which are tiny membranous vesicles that contribute to thrombogenesis and may have a pathogenetic role in different experimental and clinical conditions, including inflammatory diseases and cancer (Giacomazzi et al., 2016). Furthermore, several recent reports described the application of annexin V in the detection and quantification of certain types of exosomes (Arraud et al., 2015), especially those originated from destroyed cancer cells after treatment (Keller et al., 2009). These are small membranous vesicles (around 150 nm in diameter or smaller) secreted by most cell types, and could be present in many and perhaps all eukaryotic fluids, including blood, urine, and media of cell cultures. They might play important roles in blood coagulation, intercellular communication, and waste management (van der Pol et al., 2012). Therefore, there is a growing interest in the clinical applications of exosomes, especially as biomarkers for health and disease (van der Pol et al., 2012). As a matter of fact, the structure and mechanism of action of exosomes have formerly been exploited to a great deal in nanobiotechnology in preparing membranous nanostructures, called liposomes, for specific and secured drug delivery and cancer imaging (de Araújo Lopes et al., 2013; Zhang et al., 2014).

The commercially available annexin V conjugated to fluorochromes is used, as a molecular imaging agent, to identify and quantify the early apoptotic cells *in vitro* and in animal models and patients by fluorescent microscope and different flow cytometry (FCM) analysis (Logue et al., 2009; Lizarbe et al., 2013). The necrotic and late apoptotic cells can be excluded from viable cells using propidium iodide (PI), a fluorescent dye that intercalates into DNA and labels the nucleus (Abskharon et al., 2011). The fluorescent labeling of annexin V requires a chemical linkage with fluorescein isothiocyanate (FITC). In spite of being well-established method, labeling annexin V with FITC requires multiple manipulations of the protein and results in a heterogeneous mixture of labeled protein molecules, which vary in the number, and position of bound FITC molecules. Beside the need of precise controls for further exclusion of free fluorescein, the amine-directed chemical modification of annexin V might interfere with its membrane-binding activity (Tait et al., 2006).

Protein expression system in *Escherichia coli* is an affordable method for annexin V production in high yields since no post-translational modifications are reported in its protein structure (Yuan et al., 2004; Marder et al., 2014). The green fluorescent protein (GFP) from *Aequorea* jellyfish has become a common fusion fluorescent tag because of its interesting spectral and structural features (Waldo et al., 1999; Waldo, 2003). Recently, Waldo and coworkers reported the engineering of a superfolder GFP (*sf*GFP) that showed improved folding kinetics and increased solubility (Pedelacq et al., 2006; Andrews et al., 2007; Fisher and DeLisa, 2008; Wu et al., 2009). Enhanced characteristics were observed in many proteins when fused to *sf*GFP, proving the usefulness of this tag as a mean to improve protein expression, detection and purification (Cabantous et al., 2005; Cabantous, 2006). Similarly, *E. coli* was found to be an ideal host for expressing soluble and stable *sf*GFP at high yields, especially that the gene of *sf*GFP was codon optimized to fit perfectly with this prokaryote translation machinery (Wu et al., 2009; Al-Homsi et al., 2012a).

A previously published work described the successful production of annexin V fusion with standard or enhanced GFP (EGFP) in *E. coli* (Ernst et al., 1998). In this work, we describe the preparation and characterization of a more homogenous structure of annexin V through fusion with *sf*GFP. Computational 3D structure prediction of this fusion suggested a total accessibility of annexin V moiety for PS binding, and consequently this was confirmed in our immunological experiments. Furthermore, we have found that this reagent offers superior sensitivity for PS and liposomes, which carry PS over the chemically modified fluorescein-conjugated annexin V, which have been prepared and tested in this work as well. Optimized *sf*GFP-ANXV fusion could be effectively invested as affordable, rapid, sensitive and reproducible molecular tool for PS detection on membranes of either apoptotic cells or exosomes.

## Materials and methods

### Antigens and antibodies

For ELISA and immunoblotting tests, detection of recombinant proteins was performed using a specific mouse anti-6× His monoclonal antibody (R&D biosystems). Biotinylated proteins were detected with a polyclonal anti-biotin antibody (Bethyl Laboratories Inc.). Free and fusion *sf*GFP proteins were detected using anti-GFP polyclonal antibody (Al-Homsi et al., 2012a). Subsequent detection of rabbit or mouse antisera in ELISA or immunoblotting was performed with anti-rabbit or anti-mouse conjugated to horseradish peroxidase (HRP) or to alkaline phosphatase (AP) (Bethyl Laboratories Inc.). Soluble *sf*GFP was expressed and purified from *E. coli* BL21-GOLD (DE3) (Stratagene) containing pRSET-*sf*GFP plasmid according to a previously described method (Al-Homsi et al., 2012a).

### Cloning and expression of recombinant Annexin V

Total RNA was extracted from the hepatoma cell line (HepG2) using Illustra RNAspin Mini Kit (GE Life Sciences) following the manufacturer's instructions. Two microgram of RNA were reverse-transcribed to cDNA using Ready-to-Go You-prime first-strand-beads (GE Life Sciences) with oligo-dT^15−18^ (Invitrogen). Two microgram of cDNA were used as a template in a PCR with a pair of annexin V specific primers; ANXV-F and ANXV-R (*Eco*RI/*Hind*III) (Supporting Material, Table S1). The cDNA was amplified by a high fidelity Taq DNA polymerase (AccuPrime™Kit; Invitrogen) at 55°C annealing temperature resulting in the amplification of 1031 bp DNA fragment. This fragment was cloned in pRSET-S50 (un published data) and pRSET-*sf*GFP-KMP11 (Meriee et al., 2014) plasmids, using *Nco*I and *Eco*RI restriction enzymes in order to construct pRSET-ANXV and pRSET-*sf*GFP-ANXV plasmid constructs, respectively (Supporting Material, Figure S1). Plasmid constructs were confirmed by sequencing and used to transform a freshly prepared electro-competent *E. coli* BL21-GOLD (DE3) cells by electroporation, then cells were grown in LB/Amp plate. A single positive colony from the plate was inoculated into 250 mL LB/Amp medium. The culture was grown at 37°C until the OD_600_ reached 0.5–0.7 then IPTG (isopropyl β-D-thiogalactoside, 0.1 mM, Promega) was added to the culture for the induction of protein expression, and the culture was grown overnight at 19°C.

### Purification of recombinant Annexin V

Cells from induced cultures were pelleted by centrifugation and suspended in binding buffer (20 mM Tris-base, 300 mM NaCl, 20 mM imidazole and pH 7.4) then lysed by French–Press (pressure 1.4 bar), centrifuged for 8 min at 10,000 × g to recover the supernatant containing the recombinant proteins (ANXV, *sf*GFP-ANXV, and *sf*GFP). Using fast protein liquid chromatography (FPLC) AKTAprime plus system (GE Healthcare), recombinant proteins were purified from the cytoplasmic extracts using a 5 mL nickel-charged column (GE Healthcare). After washing, bound proteins were eluted from the column using elution buffer (20 mM Tris-base, 300 mM NaCl, and 500 mM imidazole). The eluted fractions were concentrated by Vivaspin concentrators with a molecular mass cutoff of 10 kDa (Vivascience). Purified proteins concentrations were determined by Bradford method and adjusted to 1 mg/mL for prolonged storage at −20°C.

### Conjugation of recombinant Annexin V with biotin and FITC

Different proteins were labeled with biotin (Biotin N-hydroxysuccinimide ester, BNHS, Sigma) and ANXV was labeled with the fluorescein (6-[Fluorescein-5(6)-carboxamido] hexanoic acid N-hydroxysuccinimide ester, FNHS, λ_ex_: 490 nm/λ_em_: 514 nm, Sigma), using standard procedure for amine coupling provided by the manufacturer's instructions. The fluorescence, expressed as a relative fluorescent unit (RFU), of the two *sf*GFP derivatives was monitored by measuring it at different pairs of wavelengths for excitation (λ_ex_) and emission (λ_em_) using Fluoroskan Ascent® microplate reader (Thermo Labsystems), and the free fluorophore FNHS was used as a control.

### SDS-PAGE and immunoblotting

SDS-PAGE was performed using Bio-Rad mini-Protean II system following the manufacturer's instructions. Gels were prepared using stacking and running gels 5 and 12% respectively. After electrophoresis, the gel was stained with coomassie blue for 2 h followed by destaining in 5% acetic acid and 10% methanol. For immunoblotting, separation was carried out using 0.25 μg of pure proteins, which then were blotted onto 0.45 μm nitrocellulose membranes (BioRad) using 1× blotting buffer (25 mM Tris-base, 200 mM glycine, 0.1% SDS, and 20% methanol). After blocking with PBS containing 5% skimmed milk, membranes were incubated with the indicated dilutions of the primary antibodies for 1 h at room temperature. After several washes with PBS containing 0.05% Tween-20, separated proteins were detected by an anti-6× His (1:2,000), anti-GFP (1:3,000), and anti-Biotin (1:2,000) antibodies as indicated. Finally, blots were incubated with the secondary antibodies conjugated to AP (1:2,000) for 1 h at room temperature. Band revelation was achieved by adding chromogen substrate (0.05% NBT and 0.025% BCIP; Sigma) in AP buffer (100 mM tris-base, 100 mM NaCl, 5 mM MgCl_2_, pH 9.5).

### PL extraction and detection by ELISA and dot blotting

Phospholipids were extracted from 1 g of soya bean (~30 mg PL) or egg yolk (~100 mg PL). Tissues were homogenized in 4 mL methanol before adding 8 mL chloroform then mixing for 30 min on a rotary shaker at 4°C in glass tubes. After centrifugation at 800 × g for 3 min, solvents were filtrated on blotting paper and washed with 2.5 mL NaCl 0.9%. Another centrifugation at 800 × g for 3 min was required to separate the phases before eliminating all of the upper inorganic phase. Finally, PL was dried from chloroform under a stream of N2 for 1 h, then in vacuum rotation overnight. Phospholipids (30 μg/mL) were immobilized onto the wells of polystyrene microtiter plates (TPP) or PVDF membrane (BioRad) in methanol: chloroform mixture (4:1). Wells or membranes were blocked with 5% skimmed milk in HBS (10 mM Hepes, 150 mM NaCl, 2.5 mM CaCl2, pH 7.4), washed with HBS and then allowed to bind purified and biotinylated ANXV, *sf*GFP-ANXV, and *sf*GFP (5 μg/mL) in HBS for 1 h at 22°C. After washing with HBS, bound proteins were detected with anti-biotin or anti-GFP antibodies.

### Liposomes preparation

Dried PL were suspended in HBS at a final PL concentration of 5 mM (~3.75 mg/mL, MW: ~775). Sonication was performed in ice for 2 min, pulse 15 s/repose 45 s and amplitude 35% using 750 Watt Ultrasonic Processor (Cole Parmer). Then the mixture was centrifuged at 10 k × g for 30 min. Finally, the milky supernatant was filtrated five times through 0.22 μm syringe filter. The size of the prepared liposomes was analyzed using Scanning Electron Microscope (SEM). Liposomes samples were coated with carbon using (K975X turbo evaporator), then the coated samples were observed and examined with the help of SEM and photographs were taken of different liposomes sources.

### Surface plasmon resonance (SPR)

Annexin V binding to PL was investigated using SR7000DC biosensor (Xantec). Phospholipid liposomes (0.1 mM) in HBS-T buffer (0.005% Tween-20) were passed over flow cells of a research grade biosensor chip coated with phytosphingosine (Xantec) at a flow rate of 50 μl/min forming a lipid bilayer surface according to the manufacturer's instructions. The target amount of immobilization was 1,000 response units (RU). The high binding capacity of liposomes was demonstrated by consecutive injection of five aliquots (100 μl) of liposomes (100 μM) over the left flow cells and followed by a dissociation step of 200 s. All binding experiments were performed at 25°C in HBS-T buffer at a flow rate of 50 μl/min. ANXV, *sf*GFP-ANXV, and *sf*GFP, were diluted in HBS-T buffer to obtain concentrations of 100 nM. Then they were injected over immobilized PL and control channels for 120 s (association phase), followed by a dissociation phase of 240 s (buffer alone). The sensorgrams were fitted by subtracting the signal from the reference flow cell and were globally treated using Scrubber 2 software (www.cores.utah.edu). The cells were regenerated with 5 mM EDTA in HBS-T between cycles.

### Cell culture and Annexin V binding assay

HeLa cells were cultured in RPMI 1640 medium supplemented with 10% fetal bovine serum (FBS), and 100 U/mL of penicillin and 100 mg/mL of streptomycin (all from Sigma Chemical). Apoptosis was induced in HeLa cells (10^6^ cell/mL) by various stimuli as indicated. Annexin V binding assays were performed using Annexin-V-FITC Apoptosis Kit (CLONTECH Laboratories), following the manufacturer's instructions. Similarly, FNHS-ANXV, *sf*GFP-ANXV, and *sf*GFP proteins (2 μg/mL) were used for cell staining by incubation for 15 min in the dark. Apoptotic cells were identified either by direct visualization of green-colored membrane staining under a fluorescent microscope or by flow cytometry. To distinguish cells that had lost membrane integrity, propidium iodide (PI) was added at final concentration of 2 μg/mL before analysis.

### Flow cytometry and fluorescent microscopy

Liposomes (0.1 mM) and PI/annexin V stained HeLa cells (10^6^ cell/mL) were diluted with HBS before acquisition on a BD FACS Calibur™ flow cytometer (BD Biosciences) with a 488 nm argon ion laser and a 635 nm red diode laser. The light scatter and fluorescent parameters were set at logarithmic gain, and platelets were identified according to their characteristic forward and side scatter properties, with 10,000 total events per sample acquired. Flow cytometric data acquisition and analysis were conducted by BD Cellquest™ Pro software. UV-treated HeLa cells were analyzed using a fluorescent microscope (AxioImager.Z1 mot, Carl Zeiss Ltd.), and an ISIS imaging system (MetaSystems) was used for images capturing and processing.

## Results

### Designing of *sf*GFP-ANXV fusion protein

**Figure 1:**
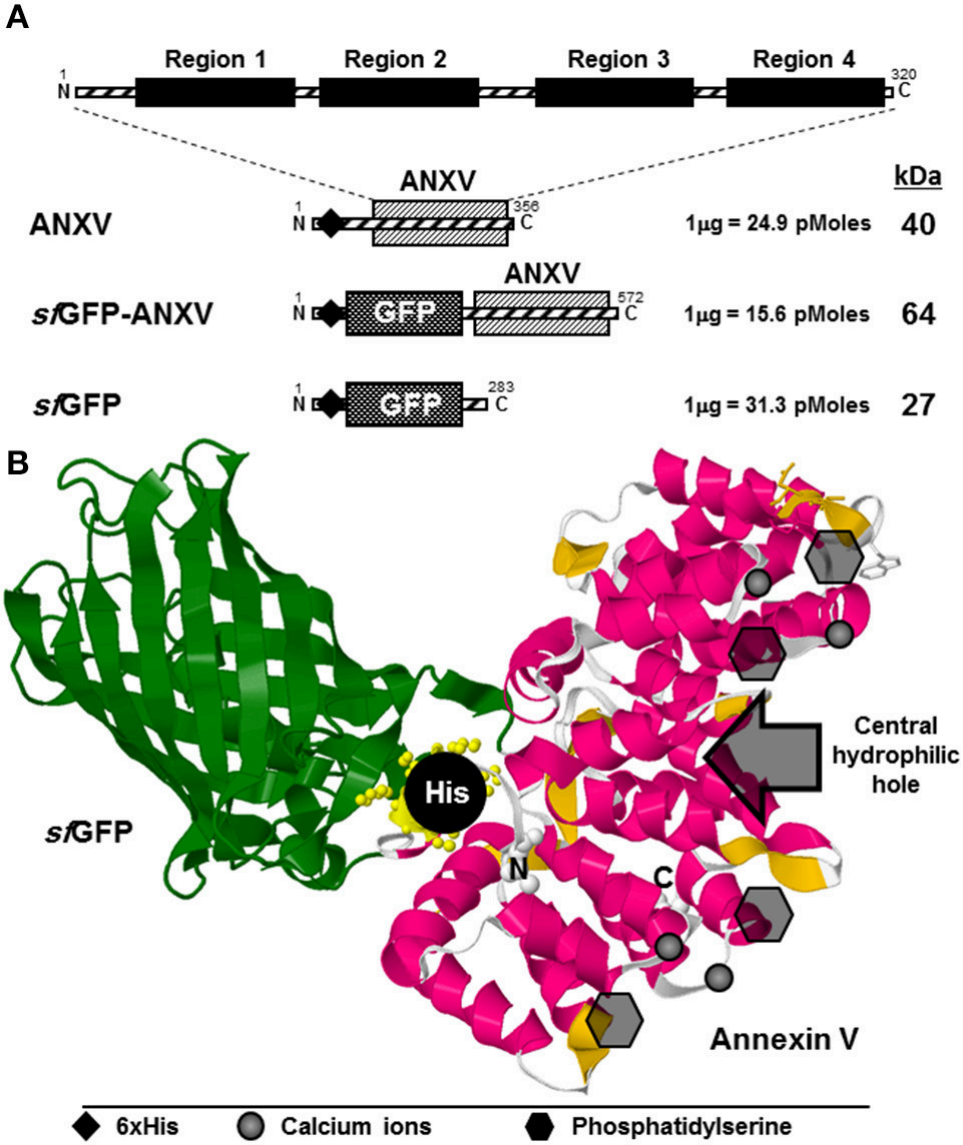
**Designing of ANXV in free and *sf*GFP-fusion forms**. **(A)** Schematic representation of annexin V beside the derivative recombinant proteins; ANXV, *sf*GFP-ANXV, and *sf*GFP, used in this study. The theoretical molecular size (kDa) and molecular weight (pMoles/μg) are shown to the right of each recombinant construct. Positions of the 6× His tag are shown using ♦ symbol. **(B)** Cartoon representation of the modeled 3D structure of *sf*GFP-ANXV fusion, where the calcium binding sites, PS sites, central hydrophilic cavity, and the N-terminal 6× His tag are shown. Structure simulation was predicted using Phyre2 server (Kelley et al., 2015).

Human annexin V is a relatively small protein of 35 kDa composed of 320 amino acids (aa), extending over four functional and well identified domains (Figure 1A). Each of these homolog domains extends over 61 aa forming a conical structure responsible for PS binding in the presence of calcium ions. Annexin V is characteristically devoid of potential glycosylation sites and disulphide bridges, and so is *sf*GFP, making their expression in a reducing environment (like *E. coli* cytoplasm) suitable. The human gene for annexin V was cloned in two different plasmids; pRSET-a and pRSET-*sf*GFP downstream *sf*GFP gene, resulting in N-terminal 6× His-tagged proteins with theoretical sizes of 40 and 64 kDa, respectively (Figure 1A). The original plasmid pRSET-sfGFP was used for the expression of N-terminal 6× His-tagged *sf*GFP of 27 kDa in *E. coli* (Figure 1A; Al-Homsi et al., 2012a; Twair et al., 2014). As inferred from the 3D structure prediction of the fusion protein *sf*GFP-ANXV, the PS binding side of annexin V appeared to be exposed away from *sf*GFP and in a favorable direction for binding PS-displaying membranes (Figure 1B, Supporting Material, Data Sheet 1). In addition, the N-terminal 6× His tag seemed to be laterally exposed and suitable for protein purification by nickel affinity chromatography. A similar favorable structure was predicted for the protein ANXV despite the existence of a relatively long sequence (36 aa), initiated with the 6× His tag, at its N-terminal side (Supporting Material, Data Sheet 2).

### Expression of fluorescent *sf*GFP-ANXV fusion protein

**Figure 2:**
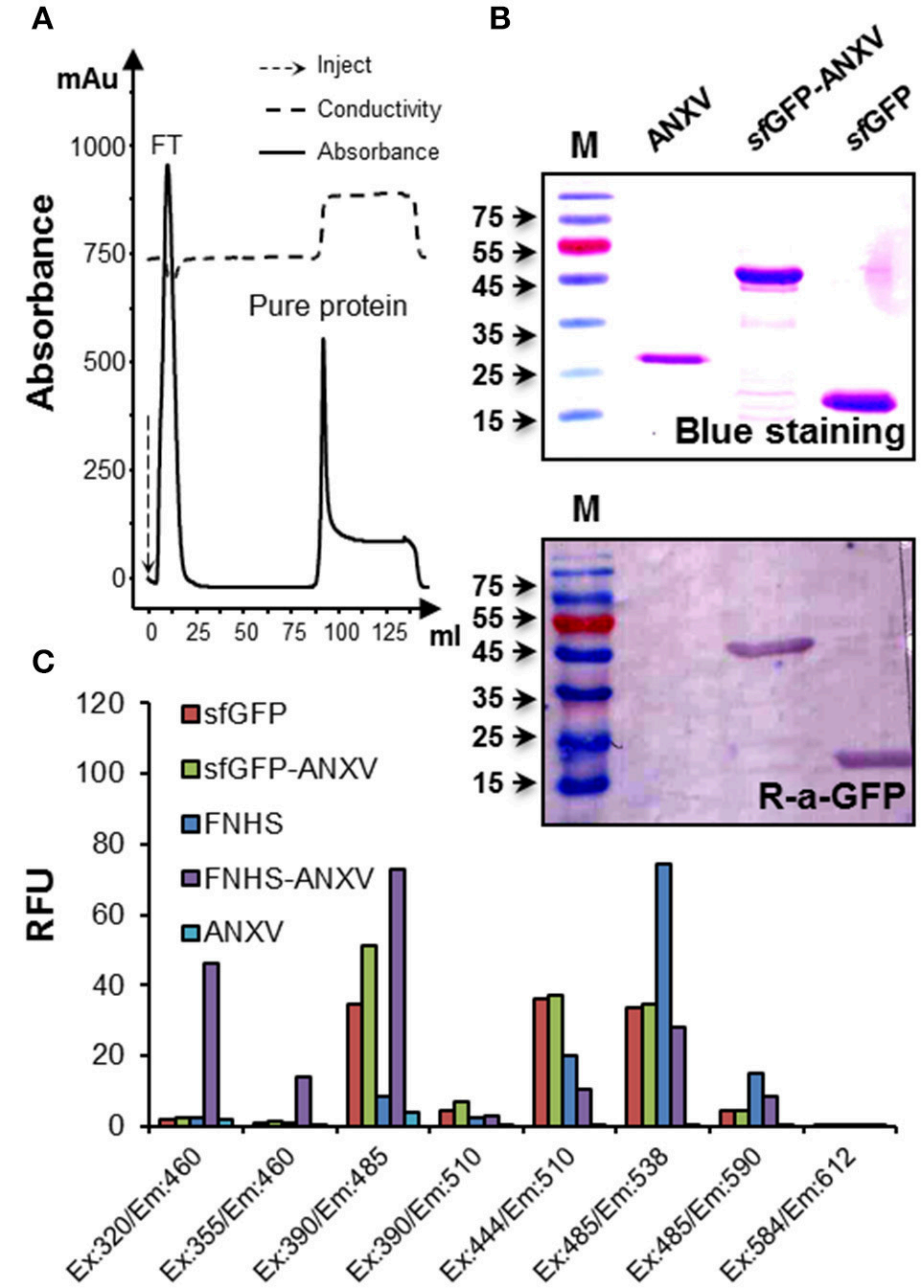
**Purification and characterization of *sf*GFP-ANXV**. **(A)** Diagram of purification procedure using nickel-charged column installed on FPLC AKTA prime system. Continuous line represents the absorbance of the eluate, and the peaks of the flow-through sample and purified protein (*sf*GFP-ANXV) are indicated. Dashed line represents the conductivity of the eluate. **(B)** Detection of the purified ANXV, *sf*GFP-ANXV, and *sf*GFP was done after SDS-PAGE separation either by blue staining or by immune blotting using anti-GFP antibodies. The protein molecular weight ladder is in the first lane (M). **(C)** Fluorescence spectra of the different proteins (30 μg/mL) and the fluorophore fluorescein FNHS (1 μg/mL) were determined by measuring at available pairs of wavelengths on the Fluoroskan Ascent® microplate reader. The values were expressed as a relative fluorescent unit (RFU). Blank conditions represent the fluorescence of unlabeled ANXV-containing wells.

Expression of *sf*GFP-ANXV was carried out after transformation of *E. coli* BL21 (DE3) Gold cells with the confirmed pRSET-ANXV and pRSET-*sf*GFP-ANXV constructs. A remarkable expression of soluble proteins was observed from both constructs after IPTG induction, and a brief amount of the proteins was found insoluble (data not shown). Furthermore, the 6× His tag of both proteins, ANXV and *sf*GFP-ANXV, was functional in affinity purification on a nickel-charged column (Figure 2A). Expectedly, the real sizes of both proteins and the control *sf*GFP were identical to their theoretical ones as shown on SDS-PAGE after blue staining (Figure 2B). Beside their clear greenish color, GFP-containing recombinant proteins (*sf*GFP and *sf*GFP-ANXV) were specifically detected by immunoblotting with an anti-GFP antibody (Figure 2B). ANXV was covalently linked to the fluorescein fluorophore (FNHS) through amine conjugation, and the fluorescence characteristics of *sf*GFP-ANXV and FNHS-ANXV were investigated and compared with *sf*GFP as well as with the free fluorophore. Apparently, fluorescence spectra of the free and fusion *sf*GFP seemed to be identical at several pairs of wavelengths for excitation and emission and differ from the spectrum of the fluorescein. On the contrary, chemical conjugation resulted in a remarkable spectrum shifting between the free and bound FNHS, from λ_ex_: 485/λ_em_: 538 (advised by the supplier λ_ex_: 490 nm/λ_em_: 514 nm) to λ_ex_: 390/λ_em_: 485 (Figure 2C).

### Annexin V binding to phospholipids

**Figure 3:**
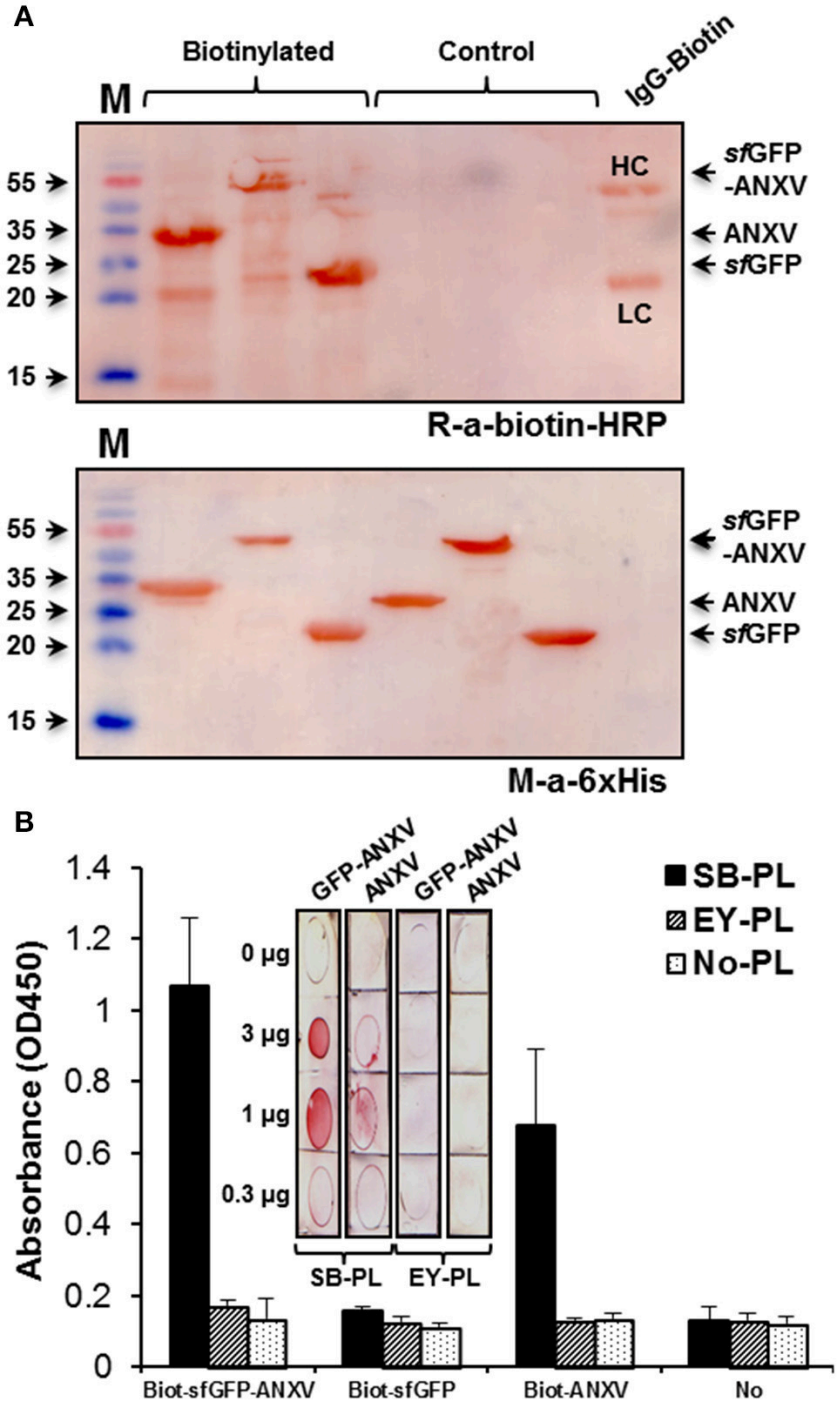
**Testing of annexin V binding with phospholipids**. **(A)** SDS-PAGE separation of ANXV, *sf*GFP-ANXV, and *sf*GFP with and without labeling with biotin-NHS. Immunoblotting was done using anti-6× His and anti-biotin antibodies. Biotinylated IgG antibody (IgG-Biotin) was used as a control where it's heavy (HC) and light (LC) chains are shown. **(B)** Biotin-labeled antigens were tested in ELISA for binding with the immobilized phospholipids (PL) prepared from soya bean (SB) and egg yolk (EY). Bound antigens were detected using rabbit anti-biotin antibody. (Inset) Dot blotting of different quantities of SB and EY phospholipids on PVDF membranes followed by detection with biotinylated *sf*GFP-ANXV or ANXV then with an anti-biotin antibody.

The capacity of the two purified recombinant forms of annexin V to bind phospholipids from two natural sources; soya bean and egg yolk, was tested by indirect ELISA. To unify the detection method of the different recombinant proteins after binding to the immobilized layer of phospholipids, they were all conjugated with biotin-NHS, and conjugation was confirmed by western blot using anti-biotin-HRP (Figure 3A). Detection of these different proteins in the immunoblotting was also possible using anti-6× His antibody. However, ELISA detection signal using this antibody was extremely low (data not shown). Interestingly, *sf*GFP-ANXV was able to interact with the immobilized PL from soya bean, but not from egg yolk, and ANXV interacted as well, but to a lesser extent, to soya bean PL (Figure 3B). This interaction was demonstrated and confirmed alternatively, by spotting different amounts of the phospholipids (from soya bean or egg yolk) on PVDF membranes before being detected by biotinylated *sf*GFP-ANXV or ANXV (Figure 3B, inset).

### Annexin V binding with liposomes

**Figure 4:**
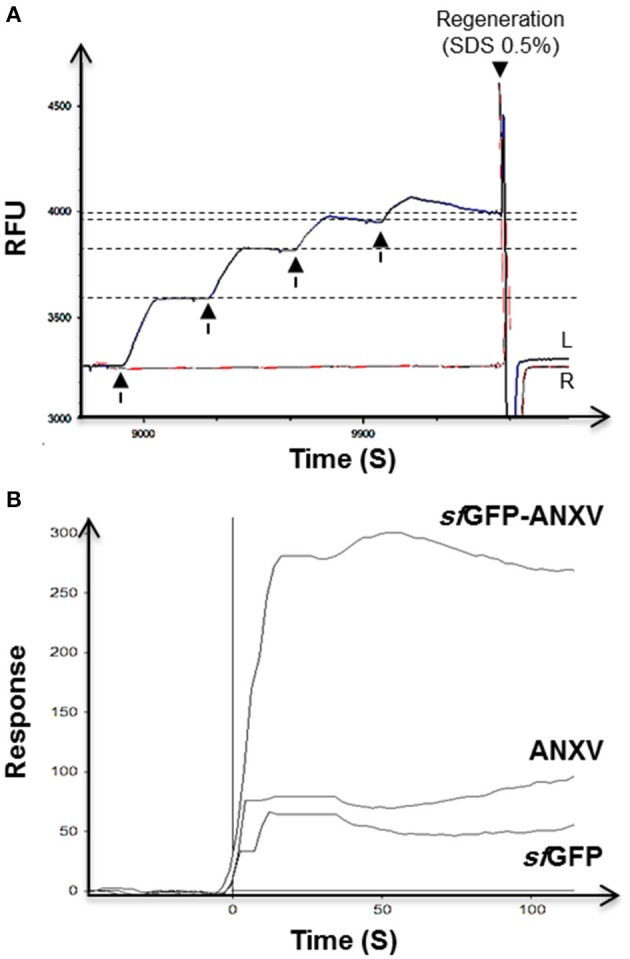
**SPR test of annexin V binding with phospholipid's liposomes. (A)** Double channels SPR sensorgram of the immobilization of four consecutive injects (arrows) of soya bean liposomes (100 μl) in the mobile phase through the left channel (L) on a sensor chip with phytosphingosine-treated surface, and the right channel was left as reference (R). Surface regeneration was achieved using a current (50 μl) of 0.5% SDS. **(B)** SPR interaction sensorgram of *sf*GFP-ANXV, ANXV, and *sf*GFP injected over the captured liposomes. SPR values were corrected for signal from control channel lacking immobilized liposomes.

The difference between *sf*GFP-ANXV and ANXV in binding to PL from soya bean was demonstrated by SPR after preparing liposomes from these PL. A special sensor chip of phytosphingosine surface immobilized on a 2D carboxymethyldextran hydrogel was used to immobilize the liposomes in a similar way to what have previously been reported (Kim et al., 2015). The binding capacity of the surface was investigated when consecutive pulses of liposomes were injected over the left channel of the sensor chip installed on a dual-channel biosensor. After each injection, a new RFU level was reached and stabilized, then finally the immobilized liposomes were washed away and the initial base line was reconstituted by injecting SDS (Figure 4A). Over the immobilized liposomes, pulses of the three different proteins were injected and the signal was normalized for each protein with the signal from the right channel. Interestingly, only *sf*GFP-ANXV was able to bind to the immobilized liposomes and not ANXV or *sf*GFP (Figure 4B).

**Figure 5:**
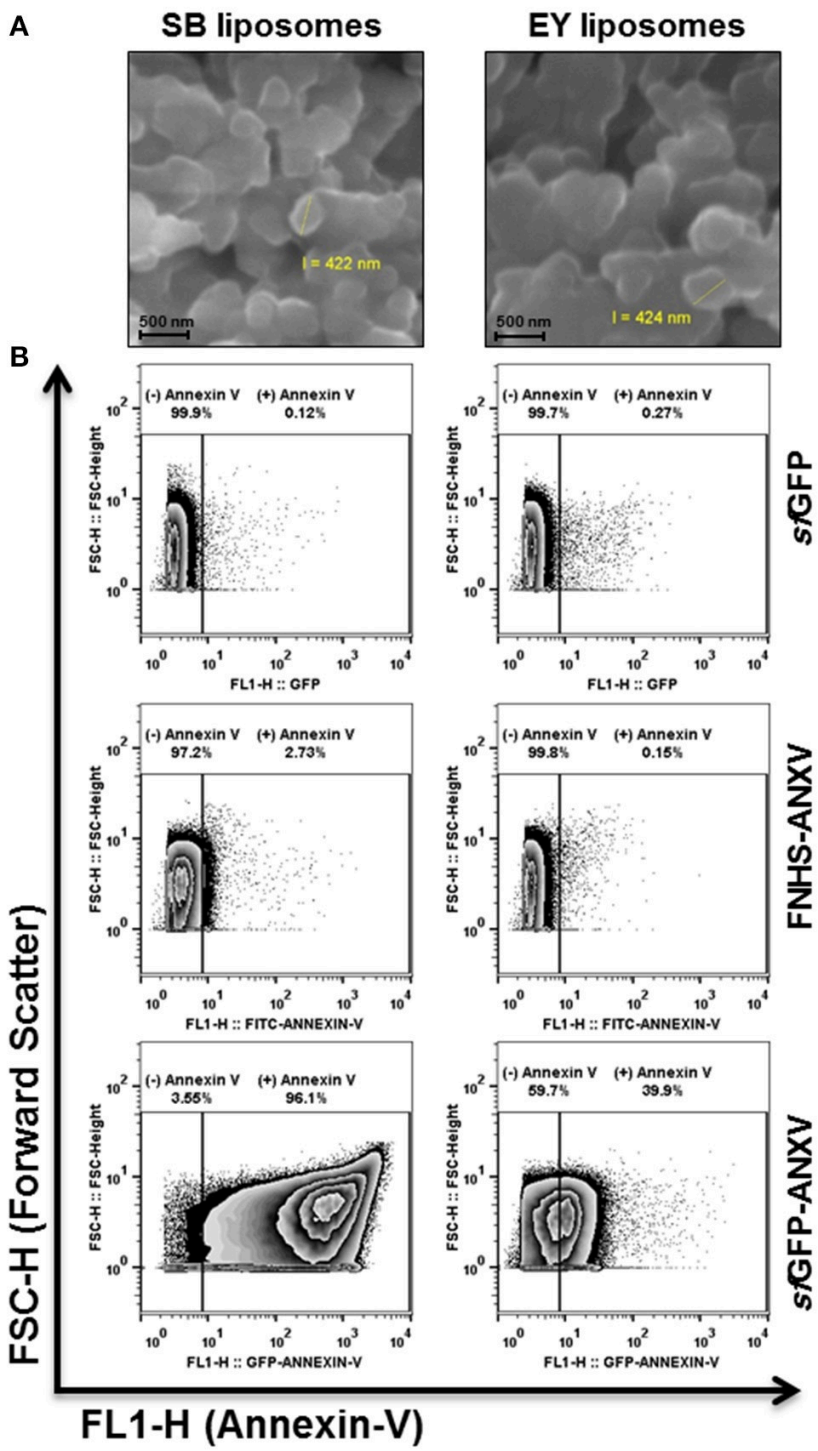
**Liposomes tagging with annexin V and scanning by flow cytometry. (A)** SEM images of soya bean (SB) and egg yolk (EY) liposomes after carbon coating, all scale bars are 500 nm. **(B)** Liposomes (10^5^ particles/mL) were prepared using phospholipids from soya bean and egg yolk, labeled either with *sf*GFP, FNHS-ANXV, or *sf*GFP-ANXV before running by flow cytometer. The size (forward scatter FSC-H) and the fluorescence (FL1-H) of liposomes population in each condition were presented. Percentages of annexin V positive (+) and negative (−) populations are shown above each panel.

Prepared liposomes from soya bean and egg yolk PL were seen under the scanning electronic microscope as microspheres of ~400 nm (Figure 5A). The differential capacity of liposomes detection between *sf*GFP-ANXV and ANXV was tested also by flow cytometry, where liposomes, from soya bean and egg yolk, were stained with each of the three recombinant proteins; *sf*GFP-ANXV, FNHS-ANXV, and *sf*GFP (Figure 5B). Interestingly, significant annexin V positive populations were observed in just three combinations; FNHS-ANXV with soya bean liposomes, *sf*GFP-ANXV with liposomes from soya bean and egg yolk, giving 2.73, 96.1, and 39.9% of total analyzed particles, respectively (Figure 5B).

### Apoptosis detection by Annexin V fluorescent derivatives

**Figure 6:**
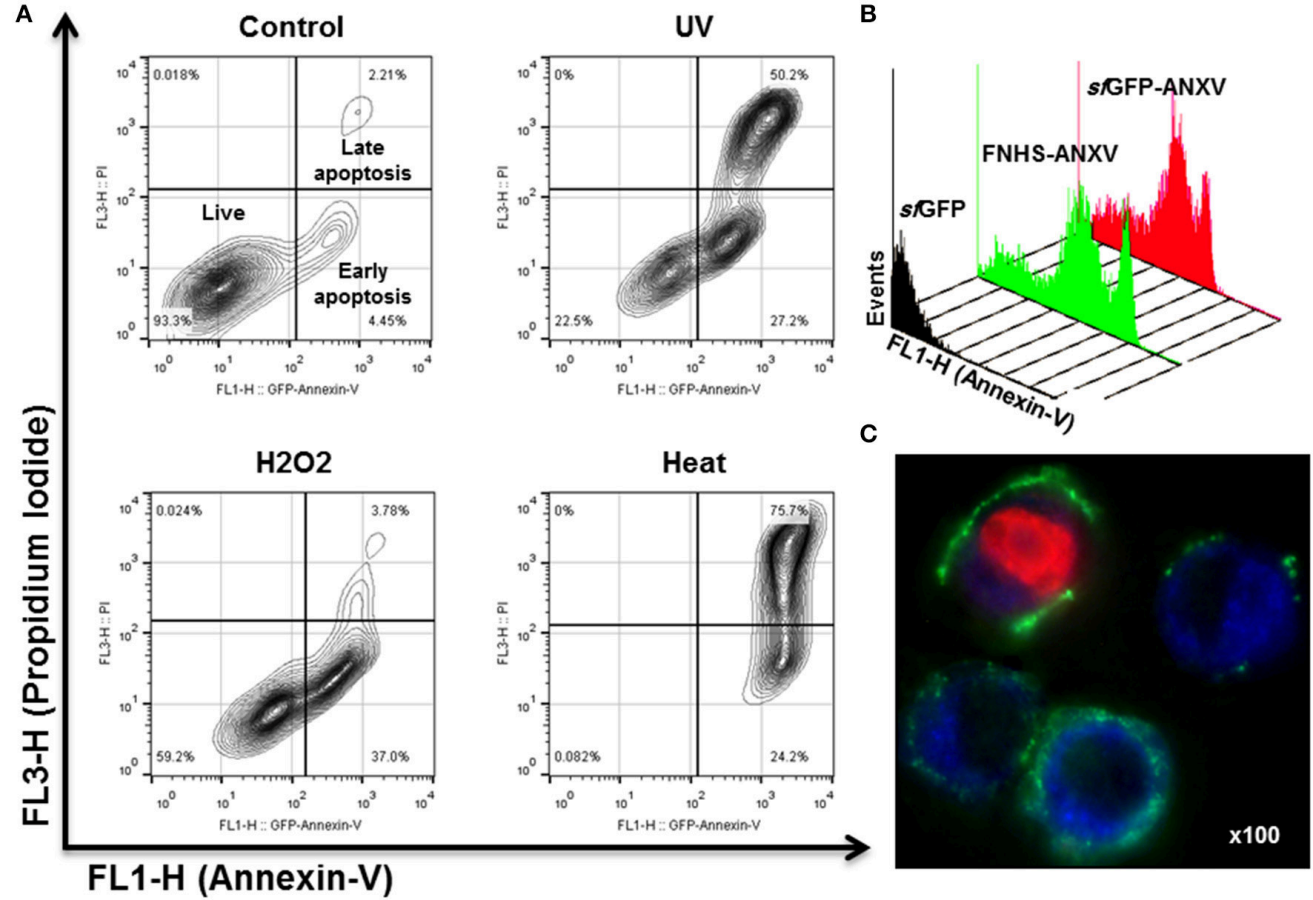
**Apoptosis detection in HeLa cells using *sf*GFP-ANXV. (A)** Flow cytometry of 10^5^ of untreated HeLa cells (control) or exposed to three different physical (heat at 55°C for 10 min or 1 J of UV light) or chemical (10 mM of H_2_O_2_) stimuli inducing cell stress and apoptosis. The PI (FL3-H) and *sf*GFP-ANXV (FL1-H) fluorescence of the cell populations were presented for each condition. Percentages of living, early (annexin V positive only) or late (PI and annexin V positive) apoptotic cell populations from total are shown. **(B)** Overlaid histograms of heat-treated HeLa cells labeled with *sf*GFP, *sf*GFP-ANXV, or FNHS-ANXV, showing events-count in function of green fluorescence (FL1-H) for each condition. **(C)** Fluorescence microscopy (100× magnify) of UV-treated HeLa cells and labeled with *sf*GFP-ANXV and PI.

The behavior of *sf*GFP-ANXV, FNHS-ANXV toward HeLa cells undergoing apoptosis after different types of stimuli (heat, H_2_O_2_, and UV) was analyzed by flow cytometry. After staining cells with PI and the fusion protein *sf*GFP-ANXV, three distinguished populations (living, early, and late apoptotic cells) could be discriminated among the cells before and after treatment (Figure 6A). Living cells (93.3%) in control condition were partially displaced into early-apoptotic cells (37%) in H_2_O_2_ treated cells. UV radiation resulted in the three distinct populations; living (22.5%), early (27.5%) and late (50.2%) apoptotic cells. In heat-treatment, most of the cells undergo late-apoptosis (75.7%). Identical histograms of fluorescence of UV-treated cells were found after staining with *sf*GFP-ANXV or FNHS-ANXV but not with *sf*GFP (Figure 6B). Finally, fluorescent microscopy of UV-treated cells after staining with *sf*GFP-ANXV and PI has shown three different types of cell; uncolored cells, cells with different degrees of green-stained membrane and a third type of cells with red colored nucleus and green dissected membrane (Figure 6C). This microscopic experiment provided an additional confirmation of the utility of *sf*GFP-ANXV in marking early and late apoptotic cells, especially when used in combination with PI.

**Figure 7:**
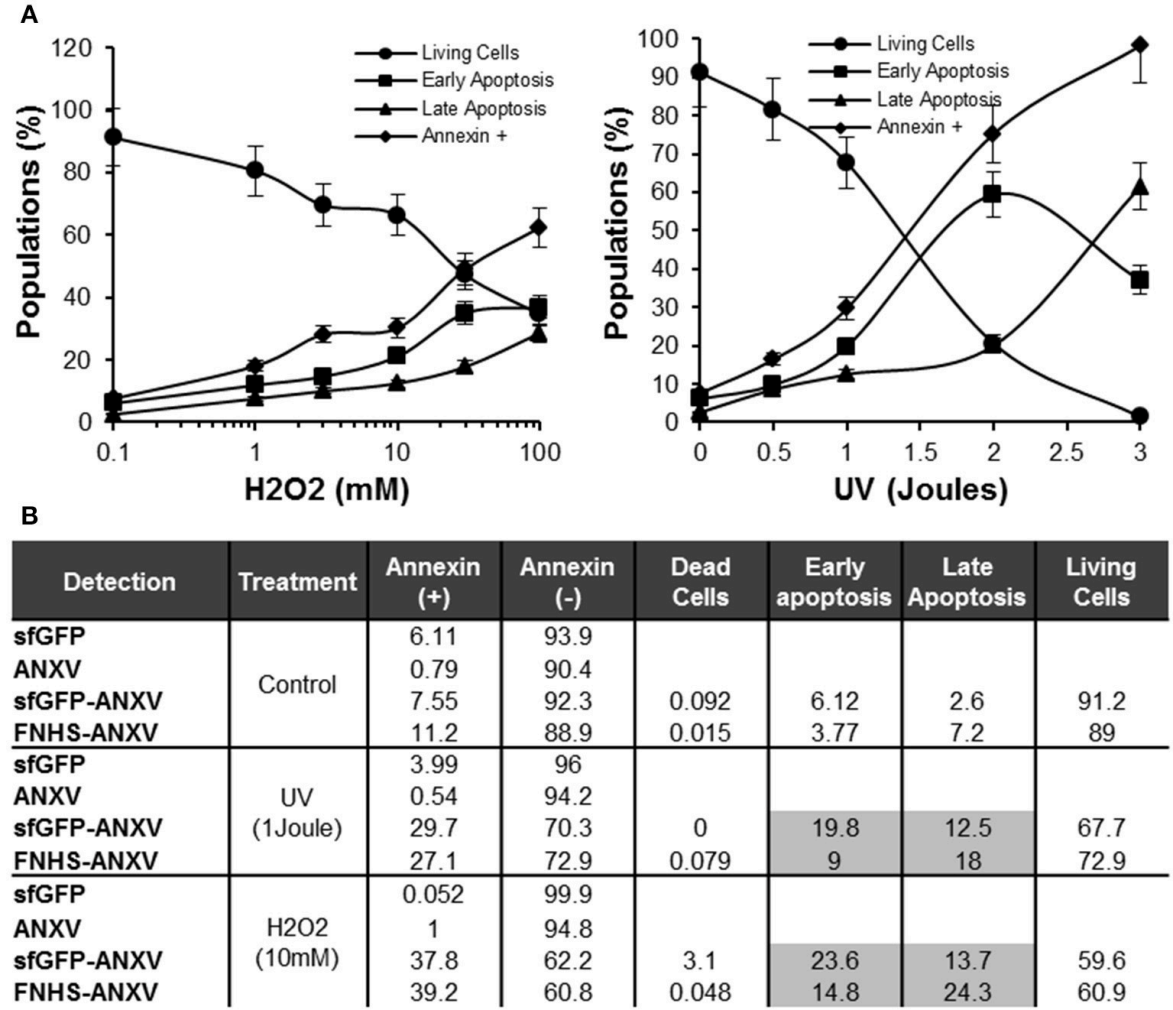
**Sensitivity of *sf*GFP-ANXV to the early apoptosis. (A)** Flow cytometry of *sf*GFP-ANXV/PI-stained HeLa cells after treatment with increasing concentrations (mM) of H_2_O_2_ or doses (Joules) of UV. Percentages of living, annexin V-positive, early (annexin V positive only) or late (PI and annexin V positive) apoptotic cell populations from total are presented in function of H_2_O_2_ concentration or UV dose. **(B)** Control HeLa cells along with the cells exposed to physical (1 J of UV) or chemical (10 mM of H_2_O_2_) stimuli inducing apoptosis, were labeled with PI and one of the recombinant proteins; *sf*GFP, ANXV, *sf*GFP-ANXV, and FNHS-ANXV before analyzing by the flow cytometry. Different cell populations were presented for each condition and staining. Range of errors of the different values in the table is expected to be <5% as calculated from at least three measurements.

The fusion *sf*GFP-ANXV was able to bind to the treated (H_2_O_2_ or UV) HeLa cells in a dose-dependent manner, and living cells decreased as annexin V-positive cells increased gradually (Figure 7A). Using H_2_O_2_ treatment, early and late apoptotic cells increased in parallel with annexin V-positive cells. Meanwhile, UV treatment with low doses has a similar effect as H_2_O_2_, but at 2 J, a huge population of early apoptotic cells appeared and directly transformed into late apoptotic cells at 3 J (Figure 7A). Using moderate doses of H_2_O_2_ (10 mM) and UV (1 J), a comparison between cell labeling with *sf*GFP, ANXV, *sf*GFP-ANXV, or FNHS-ANXV was carried on by flow cytometry (Figure 7B). As expected, negative labeling with control proteins; *sf*GFP and ANXV, did not result in a significant difference in cell percent between control and treated conditions, and most of the cells remained non-fluorescent (>90%). Fluorescent annexins: *sf*GFP-ANXV or FNHS-ANXV, have marked almost similar percentage of cells in control (7.55 and 11.2%), UV (29.7 and 27.1%), and H_2_O_2_ (37.8 and 39.2%) treatment conditions. Interestingly, separating of these marked cells between early and late apoptotic portions, because of the additional PI staining, has shown that in all conditions *sf*GFP-ANXV was accompanied with more early than late apoptotic cells (1.75 ± 0.38 fold), and vice versa in case of FNHS-ANXV (0.54 ± 0.04 fold). Despite this, the same percentage of living cells were left unstained by both fluorescent proteins in all conditions; control (91.2 and 89%), UV (67.7 and 72.9%), and H_2_O_2_ (59.6 and 60.9%; Figure 7B).

## Discussion

In our previous works, *sf*GFP was successfully applied as an innovative tag for expressing short peptides (Al-Homsi et al., 2015) and recovering growth hormone (GH) from the inclusion bodies (Abbady et al., 2014). Several anti-*sf*GFP specific nanobodies, recombinant single chain antibodies from camel, were also prepared and could have interesting applications in GFP-fusion protein technology (Twair et al., 2014). This technology represents one of the best solutions to achieve rapid, efficient, and cost-effective protein expression and purification (Southward and Surette, 2002). Many reports described the application of GFP fusions in the expression of hard-to-fold proteins (Vasiljevic et al., 2006), toxins (Soler-Jover et al., 2004), proteases (van den Berg et al., 2006) and medical short peptides (Skosyrev et al., 2003; Al-Homsi et al., 2012b) in both prokaryotic and eukaryotic cell types. Visibility, color and fluorescent characteristics of the GFP are the most distinguishable features from other fusion tags (Taubenberger and Anderson, 2007). Its unique green color (either visible blue light or UV light) allows us to monitor and follow the GFP fusion proteins through purification steps even without any measurement devices (Lee, 2009). The great advantages of GFP tag in comparison with synthetic dyes and fluorescent nanomaterials, like quantum dots, is that it can be genetically introduced into cells, tissues, or whole organisms, especially when they are needed for intracellular imaging of living cells (Choi et al., 2011).

Several studies and most available annexin V commercial products have used an FITC-conjugated form of the protein to investigate apoptotic cells (Schellenberger et al., 2004). The bioconjugation reaction of annexin V to FITC requires complicated manipulations and several purification steps. This conjugation takes 3 days in total with overnight dialysis (Logue et al., 2009) and resulted in a heterologous mixture of labeled protein molecules that vary in number and position of bound FITC molecules. Moreover, annexin V has several lysine amino acids (22 residues), many of them are in contact with the PS-binding site, thus lower levels of NH2 modification produce mixtures of unmodified and modified protein and higher levels lead usually to a loss of binding activity (Schellenberger et al., 2004) or result in quenching of the fluorescence by 40–50% upon membrane binding (Blackwood and Ernst, 1990). Therefore, bioconjugation reactionof annexin V with the different fluorochromes needs to be controlled precisely (Nazari et al., 2014).

Even though annexin V was previously expressed in a fusion form with GFP (Ernst et al., 1998; Wang et al., 2015), this work describes the first report of a functional annexin V fusion with the superfolder derivative of GFP. The most significant addition to the GFP palette in the past several years is the superfolder GFP, which possesses the two characteristic amino acid mutations of EGFP, and has unique six extra mutations at positions; S30R, Y39N, N105T, Y145F, I171V, and A206V (Pedelacq et al., 2006). *sf*GFP showed superior fluorescence activity (160% compared to EGFP) in normal and in oxidative environment like the periplasm of bacteria and the endoplasmic reticulum of eukaryotic cells (Aronson et al., 2011). It can efficiently fold even when fused to insoluble proteins and is more acid resistant than EGFP (Shaner et al., 2007). In addition, it showed increased resistance to denaturation, improved folding kinetics and increased resistance to aggregation during protein expression (Pedelacq et al., 2006). Furthermore, *sf*GFP fusions are more soluble than conventional GFP fusions and its coding sequence is optimized for *E. coli* expression system (Wu et al., 2009). Finally, *sf*GFP has proven to be very useful as a scaffold for improved protein detection and tagging both *in vivo* and *in vitro* using self-assembled *sf*GFP fragments (Cabantous et al., 2005; Cabantous, 2006).

Annexin V protein structure provides an interesting model for protein expression in *E. coli* because of the simple structure and the lack of disulfide bridges and glycosylation side chains. Computationally, we have found that the secondary structure of annexin V appeared to be maintained in the fusion form with *sf*GFP as clearly inferred from the prediction of protein 3D structure. In addition, the orientation of the functional surface of annexin V is expected to be at the opposite direction from *sf*GFP moiety, and such arrangement is favorable for PS binding. Moreover, the homogenously labeled annexin V with *sf*GFP did not affect its fluorescence properties as inferred after measuring the fluorescence of both proteins. The plasmid pRSET-a was used in this work to establish in *E. coli* a high-level protein expression, via the T7 promoter, of cytoplasmic and N-terminal 6× His tagged *sf*GFP-annexin V fusion, and thus could be efficiently purified using nickel-charged resin. The same system for annexin V purification was previously tested, where a C-terminal 6× His tag was added and successfully used to purify the protein (Wang et al., 2015). Interestingly in their model, EGFP was used and cloned downstream the annexin V gene just before the 6× His tag, and the expressed fusion protein was successfully used for the detection of apoptosis (Wang et al., 2015). Previously, Broaddus and coworkers conceived that GFP fusion preferably should be at the N-terminal side of annexin V; otherwise it might lose its capacity for PS binding (Ernst et al., 1998). However, in EGFP model a short linker was used to separate annexin V from the C-terminal fluorescent moiety (Wang et al., 2015).

In our study and in accordance with previous reports, 80–90% of annexin V (free or in fusion with *sf*GFP) was found in the soluble fraction of the *E. coli* lysate (Ernst et al., 1998). An important production yield of pure annexin V and *sf*GFP-ANXV was obtained using this system, estimated of about 150 mg/L (from about ~4 × 10^12^ cell) of bacterial culture. Besides, the manufacturing cost was considerably low because of using standard affinity chromatography procedure for purification. Different methodology is routinely used for purification of annexin V basing on its high affinity to PS in the presence of calcium ions. Purifying annexin V has long been achieved using calcium-dependent PS affinity chromatography (Ernst et al., 1998). However, in this study we choose to add an N-terminal 6× His tag to the different forms of annexin V and use it in the well-defined method of nickel-charged column to achieve the purification. The efficiency of the 6× His tag at the N-terminal of *sf*GFP in metal affinity chromatography has previously proven in different studies (Al-Homsi et al., 2012a, 2015; Abbady et al., 2014; Twair et al., 2014). The PS-affinity, even though it is laborious and expensive, has one great advantage over nickel affinity chromatography for annexin V purification in that it provides a direct proof of the functionality of the expressed protein (Ernst et al., 1998). Here, pure annexin V was tested for binding with PL prepared from different natural sources. As expected, free and fusion annexin V were able to bind PL from PS rich sources like soya bean (Liu and Ma, 2011). Egg yolk represents poor PS content thus; it was undetectable with annexin V. However, liposomes from these PLs were bound to *sf*GFP-ANXV by flow cytometry. Interestingly, only fusion-annexin V was able to detect the liposomes from this plant source. Apparently, fusion with *sf*GFP had enhanced the folding of annexin V and made it more sensitive to detect low PS-content membranes or membranes with PS from plant sources, but this conclusion needs more investigations. Many studies have demonstrated that annexin V staining method could be used to detect apoptotic cells, in various settings, without regard to the stimulus used to trigger apoptosis or the lineage of the cells under study (Martin et al., 1995; Vosjan et al., 2011). Here, HeLa cells undergoing apoptosis after different types of stimuli (heat, H_2_O_2_, and UV) was used to show the performance of the fusion ANXV protein. Our *sf*GFP-ANXV fusion has showed more sensitivity to early apoptotic cells than FNHS-ANXV.

## Conclusions

In conclusion, annexin V was produced in a fusion with an enhanced form of GFP using an efficient *E. coli* protein expression system. After affinity purification, *sf*GFP-ANXV fusion protein showed superior interaction activity with PLs, their nanostructure liposomes, and with early apoptotic cells, compared with the chemically modified ANXV with fluorescein. In fact, this ready-to-use bi-functional and structurally enhanced fusion protein, *sf*GFP-ANXV, is a valuable tool not only for diagnosing liposomes, similarly exosomes, and apoptosis, but also for studying annexin V interaction with PS and assessing its protective role for the integrity of the membrane leaflets.

## Authors contributions

AA: led the work, designed all experiments and wrote the manuscript. AT: carried out experiments and participated in writing the manuscript. BA: carried out cloning and expression experiments, HM: carried out cell experiments, beside reading, and commenting the manuscript. All authors read and approved the final version of the manuscript

## Funding

This work was funded by Atomic Energy Commission of Syria.

### Conflict of interest statement

The authors declare that the research was conducted in the absence of any commercial or financial relationships that could be construed as a potential conflict of interest.

## Supplementary material

Figure S1 **Structure of pRSET-*sf*GFP-ANXV and pRSET-ANXV plasmids**. Map of the plasmid construct pRSET-*sf*GFP-ANXV, in which the inserted ANXV is indicated. The most important elements of the plasmid are shown, including the T7 promoter, N-terminal 6× His tag, two restriction sites (*Nco*I/*Eco*RI) used for insert ligation, ampicillin resistance gene (Amp), f1/PUC origin of replication, and *sf*GFP gene.

Table S1 **The primers used for the amplification and cloning of the ANXV**. The different parameters (name, length and sequence) of the primers used for *ANXV* gene amplification and cloning into pRSET and pRSET-*sf*GFP plasmids are indicated in the table.

Data Sheet 1 **The 3D structure of sfGFP-ANXV fusion protein**.

Data Sheet 2 **The 3D structure of ANXV protein**.

## Abbreviations

ANXV: annexin V
ELISA: enzyme−linked immunosorbant assay
FPLC: fast protein liquid chromatography
*sf*GFP: superfolder green fluorescent protein
IPTG: isopropyl β-D-thiogaldctoside
PI: probidium iodide
FNHS: 6-[Fluorescein-5(6)-carboxamido]hexanoic acid N-hydroxysuccinimide ester
FITC: fluorescein isothiocyanate.
