# Supplementary figures and images for: Characterization of Annexin V Fusion with the Superfolder GFP in Liposomes Binding and Apoptosis Detection
Source: Front Physiol. 2017 May 19;8:317. doi: 10.3389/fphys.2017.00317 (PMC5437369; doi:10.3389/fphys.2017.00317)

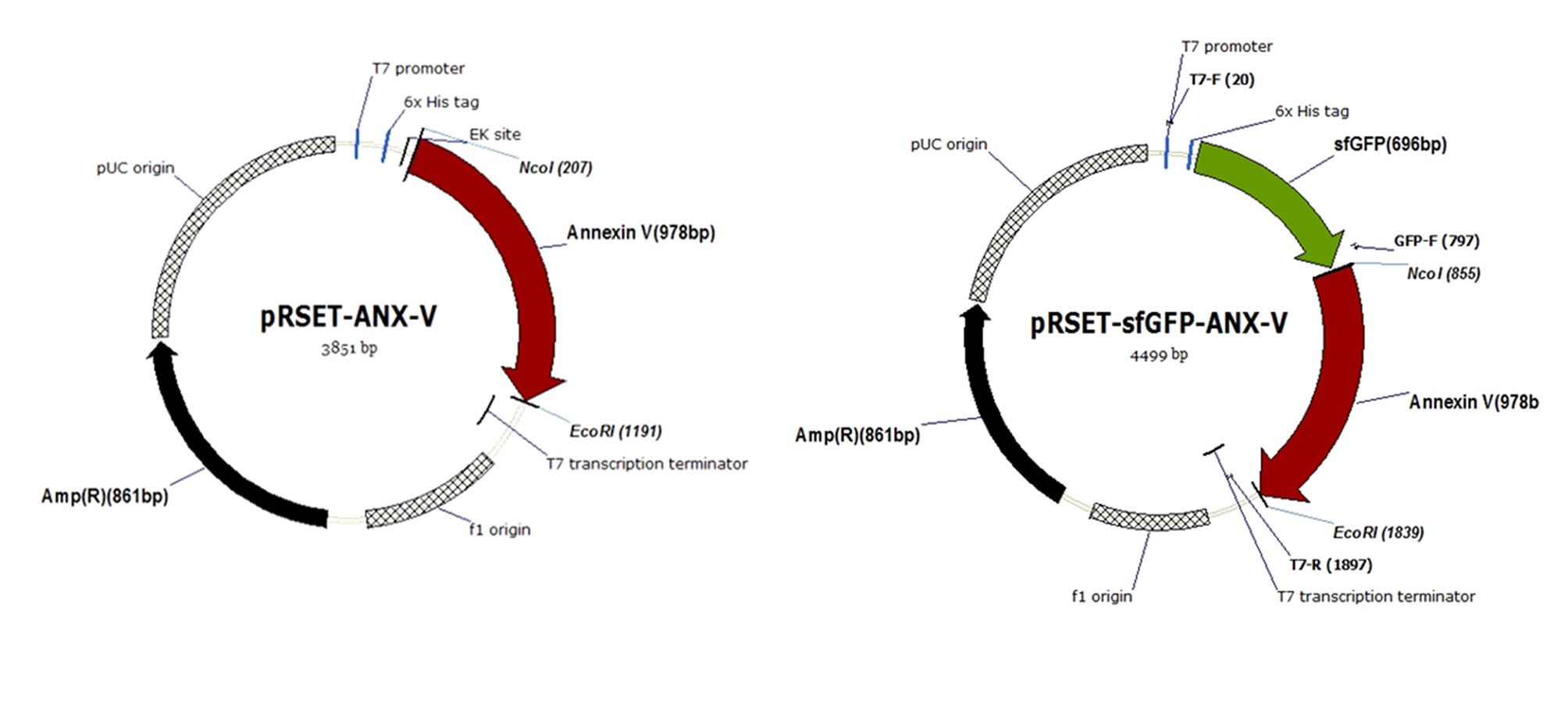

Supplement: Figure S1 — Structure of pRSET-sfGFP-ANXV and pRSET-ANXV plasmids. Map of the plasmid construct pRSET-sfGFP-ANXV, in which the inserted ANXV is indicated. The most important elements of the plasmid are shown, including the T7 promoter, N-terminal 6× His tag, two restriction sites (NcoI/EcoRI) used for insert ligation, ampicillin resistance gene (Amp), f1/PUC origin of replication, and sfGFP gene. [file Image1.TIF]

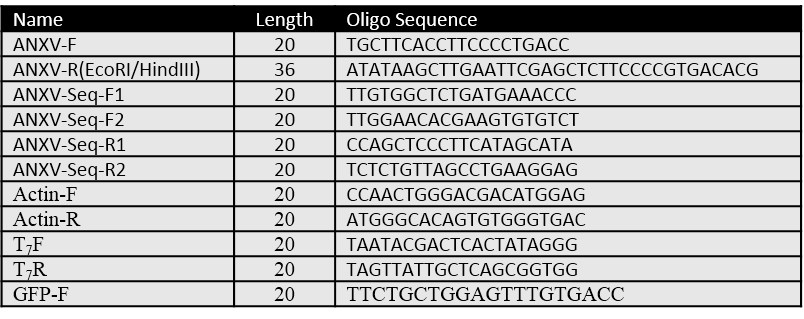

Supplement: Table S1 — The primers used for the amplification and cloning of the ANXV. The different parameters (name, length and sequence) of the primers used for ANXV gene amplification and cloning into pRSET and pRSET-sfGFP plasmids are indicated in the table. [file Table1.docx]
